# Health solutions to improve post-intensive care outcomes: a realist review protocol

**DOI:** 10.1186/s13643-018-0939-7

**Published:** 2019-01-08

**Authors:** A. Fuchsia Howard, Leanne Currie, Vicky Bungay, Margaret Meloche, Robert McDermid, Sarah Crowe, Andrea Ryce, William Harding, Gregory Haljan

**Affiliations:** 1 Faculty of Applied Sciences, School of Nursing, University of British Columbia, T201-2211 Wesbrook Mall, Vancouver, British Columbia V6T 2B5 Canada; 2 Fraser Health Authority, Surrey, British Columbia Canada; 3 Faculty of Medicine, University of British Columbia, Vancouver, British Columbia Canada; 4 Provincial Health Services Authority, Vancouver, British Columbia Canada

**Keywords:** Realist review, Intensive care, Health services research, Critical care, Rehabilitation medicine, Literature review, Post-intensive care syndrome

## Abstract

**Background:**

While 80% of critically ill patients treated in an intensive care unit (ICU) will survive, survivors often suffer a constellation of new or worsening physical, cognitive, and psychiatric complications, termed post-intensive care syndrome. Emerging evidence paints a challenging picture of complex, long-term complications that are often untreated and culminate in substantial dependence on acute care services. Clinicians and decision-makers in the Fraser Health Authority of British Columbia are working to develop evidence-based community healthcare solutions that will be successful in the context of existing healthcare services. The objective of the proposed review is to provide the theoretical scaffolding to transform the care of survivors of critical illness by a synthesis of relevant clinical and healthcare service programs.

**Methods:**

Realist review will be used to develop and refine a theoretical understanding of why, how, for whom, and in what circumstances post-ICU program impact ICU survivors’ outcomes. This review will follow the recommended five steps of realist review which include (1) clarifying the scope of the review and articulating a preliminary program theory, (2) searching for evidence, (3) appraising primary studies and extracting data, (4) synthesizing evidence and sharing conclusions, and (5) disseminating and implementing recommendations.

**Discussion:**

This realist review will provide a program theory, encompassing the contexts, mechanisms, and outcomes, to explain how clinical and health service interventions to improve ICU survivor outcomes operate in different contexts for different survivors, and with what effect. This review will be an evidentiary pillar for health service development and implementation by our knowledge user team members as well as advance scholarly knowledge relevant nationally and internationally.

**Systematic review registration:**

PROSPERO CRD42018087795

**Electronic supplementary material:**

The online version of this article (10.1186/s13643-018-0939-7) contains supplementary material, which is available to authorized users.

## Background

An increase in the use of intensive care unit (ICU) services over the past two decades, coupled with tremendous strides in the diagnosis and treatment of life-threatening conditions, like multiple organ system failure, respiratory failure, sepsis, and shock, has resulted in growing numbers of ICU survivors. Currently, up to 80% of patients will survive treatment in an ICU [1–3]. Yet, the cost of survival can be tremendous. Approximately 25 to 50% of survivors will suffer a constellation of new or worsening physical, cognitive, or psychiatric complications, termed post-intensive care syndrome (PICS) [4–7]. Mortality is also high following ICU discharge, ranging from 26 to 63% in the first year with survivors facing a two to five times higher risk of dying when compared with age-matched population controls [8, 9]. Post-hospital discharge, ICU survivors are frequently lost in transition from hospital-based acute care to community-based care and rarely have access to programs that address PICS as well as professionals with expertise in the syndrome [10]. In a Canadian study of health services use among ICU survivors, almost 40% were readmitted to a hospital in the first 2 years, with half being readmitted multiple times [11]. Medical teams that support these patients after their discharge have little evidence to guide therapy and prevent the complications that result from the accumulation of injuries during the hospital stay [10]. There is consensus that clinical and healthcare service interventions for this high-risk population are required, but the development and implementation of models of care are in their infancy.

### Health services context in British Columbia, Canada

Though post-ICU care is essential, in British Columbia, Canada, there are no programs to address the needs of patients and families suffering from PICS. Canada has a publicly funded healthcare system wherein medically necessary hospital and physician services are covered for all residents. Yet, clinicians and decision-makers in the Fraser Health Authority of British Columbia recognize that a significant number of ICU survivors with complex health issues are living in the community, frequently re-hospitalized, and in desperate need of support. These knowledge user team members contend with the consequences of limited health services for post-ICU survivors and are working to develop community healthcare solutions that will be successful in the context of existing primary and acute care services. To do so, our collaborative team of researchers and knowledge users identified an urgent need to transform the care of survivors of critical illness by conducting a synthesis of relevant clinical and healthcare service interventions that will form a core component of a new integrated community service.

### Post-ICU literature

The literature on clinical and health service interventions to improve ICU survivor outcomes is often contradictory, and existing reviews are insufficient to provide adequate evidence required by our knowledge user team members, who are developing comprehensive services. Prior reviews have been narrow in scope, focusing on interventions to address specific symptoms of PICS (e.g., depression [12], post-traumatic stress disorder [13], quality of life [14], physical functioning [15], polyneuropathy/myopathy [16]), or specific types of interventions (e.g., follow-up consultation [17] or exercise rehabilitation [18]). In their review of 14 randomized control trials of interventions to improve physical functioning, Calvo-Ayala et al. [19] found that the majority of studies failed to demonstrate efficacy and that only exercise/physical therapy-based interventions improved long-term physical functioning. In their review of five randomized control trials investigating post-ICU consultations, Jensen et al. [17] concluded that follow-up consultation might reduce symptoms of post-traumatic stress disorder but without affecting quality of life or other outcomes. The few reviews that assessed a variety of interventions came to similar conclusions. Mehlhorn et al. [20] reviewed 18 comparative studies on the effectiveness of rehabilitation interventions in post-ICU patients, and while they found that ICU diaries reduce post-traumatic stress disorder and that ICU follow-up clinics and self-help manuals reduce distress, positive findings were not consistent across the reviewed studies. They ultimately concluded that there is a lack of overall effectiveness of post-ICU interventions on physical and mental health. Reviews to date have included only a subset of comparative studies that focus almost exclusively on determining whether interventions work without adequate consideration of how interventions might improve ICU survivor outcomes. That is, they do not provide explanations of how ICU survivor characteristics, intervention characteristics, and healthcare settings affect patient outcomes.

As argued by others, progress in designing successful post-ICU interventions has been impeded by uncertainties about which patients require specific intervention, and the timing, intensity, duration, and nature of the application of an intervention [21]. A lack of knowledge about how post-ICU interventions work makes it difficult to select the most appropriate criteria to judge whether it works, and in the event that expected outcomes are not achieved, knowledge of the mechanisms and contexts in which interventions should work can help to identify why these outcomes were not achieved [22]. As such, a theory-driven approach that explicates how post-ICU interventions work is urgently needed to guide subsequent healthcare development and intervention research. In the absence of a more robust knowledge base in this area, knowledge users face the onerous task of attempting to design solutions without the essential theoretical scaffolding.

### Realist review

The complexity of our knowledge users’ needs and the state of the current evidence necessitates a realist review approach to create the theoretical scaffolding to support the development of post-ICU care. This approach embraces the intricacies of health and health service interventions, unpacks the mechanisms by which interventions work (or fail to work), and informs solutions to complex problems that require deeper insights into the nature of interventions and implementation contexts [22–24]. This approach focuses on the relationships between interventions, contexts, and outcomes to understand the mechanisms and conditions for a “family of programs” [24, 25]. In the context of our realist review, the family of programs is post-ICU interventions. A realist review involves carefully analyzing contexts, mechanisms, and outcome configurations with the goal of constructing explanations rather than testing program efficacy.

Realist review uses an iterative process to build explanations of how, and to what extent, various programs produce outcomes. In the preliminary phases of realist review, researchers seek to identify and build a foundational knowledge of relevant program theories. Subsequent phases are dedicated to consulting evidence from a range of sources to refine the program theories and to determine if these theories are valuable and useful [22]. Through these processes, realist review aims to create explanations that provide evidenced rationales for how and why programs produce specific outcomes in different contexts, for different individuals.

The explanations generated through realist review identify casual connections between programs and outcomes. However, realist review does not produce a predictive model or quantitative measurement of how likely a given program will produce a specific outcome. Rather, realist review centers on illuminating underlying mechanisms, with consideration to context, that produce outcomes. For this reason, realist methodology seeks to identify and unpack patterns that reveal hidden causal connections rather than solely focusing on the consistency and effectiveness of various programs on outcomes [22]. Within realist methodology, program theories explain how, why, for whom, and to what extent outcomes are achieved through the use of contexts, mechanisms, and outcome configurations [25]. Mechanisms are the driving forces behind building an explanation within realist thinking. Mechanisms are not interventions; rather, they are the underlying causal processes within programs that describe how and why changes occur with respect to the services and resources that programs provide [26]. When mechanisms operate, they do so contingent on context, and the realist reviewer focusses on identifying and explaining the patterns of contexts (C), mechanisms (M), and outcomes (O). These patterns of CMO configurations, which are often expressed with the equation: C + M = O, develop and refine program theories. Program theories often have multiple CMO configurations embedded within them that describe the processes by which the outcomes occur. And so, through a realist review, one ultimately seeks to construct evidence-based program theories that describe the conditions for program efficacy, specifically, for whom, in what circumstances, and through what mechanisms programs work by iteratively examining and synthesizing evidence from a range of sources [22].

### Review objective and questions

The objective of this review is to explain how clinical and health service interventions to improve ICU survivor outcomes operate in different contexts for different survivors, and with what effects. In line with a realist review approach, our three research questions are as follows:

(1) Through what mechanisms (how) do post-ICU interventions improve patient outcomes?

(2) Which contexts interact with mechanisms to influence intended and unintended patient outcomes from post-ICU interventions?

## Methods

As a team of researchers, knowledge users (clinicians and healthcare administrators), and a health science librarian, we will follow the steps of a realist review outlined by Pawson [24] that include (1) clarifying the scope of the review and articulating a preliminary program theory, (2) searching for evidence, (3) appraising primary studies and extracting data, (4) synthesizing evidence and sharing conclusions, and (5) disseminating and implementing recommendations.

### Step 1: Clarifying the scope of the review and articulating a preliminary program theory

As the review topic is potentially very wide-ranging, our initial priority was to refine and narrow the scope of the review via a broad-based scoping exercise. Through our immersion in the literature, we clarified the populations, interventions, and outcomes of interest as follows. We will include studies of adults who had an ICU stay and who have been discharged from hospital. We are primarily focused on patients who stayed in medical or surgical ICUs as these patient populations are most similar to patients of interest to our knowledge users. We will also include studies of ICU patients’ families and caregivers in the post-hospital discharge period. In British Columbia, Canada, ICU follow-up programs do not exist for a large majority of critically ill medical or surgical patients. Consequently, our review focuses on identifying studies that focus on ICU survivors for whom existing follow-up care does not exist or for whom current follow-up programs do not adequately meet their diverse health care needs.

We are only interested in interventions that occur post-hospital discharge and that are connected to patient outcomes. We will include interventions regardless of who delivers the intervention, where the intervention is delivered, how the intervention is delivered, or the intervention type (i.e., clinical or health service). We will consider both treatments and diagnostic tests as interventions if they are explicitly connected to outcomes. This broad conceptualization of interventions will enable our team to identify novel post-ICU interventions and incorporate these findings into our preliminary program theory. We will exclude interventions that exclusively occur in hospital and specific ICU-related treatments, like prolonged acute care or weaning, as well as disease-specific rehabilitation for stroke, myocardial infarction, amputation, burns, etc.

As for the outcome of interest, we are focused on long-term sequelae experienced by ICU survivors, their families, and caregivers due to the culmination of intensive care treatments and critical illness consequences that have profound impacts on patients’ health-related quality of life. We are interested in outcomes that occur ≥ 28 days post-hospital discharge, i.e., outcomes measurements that relate to survivorship. We will not limit our review to studies that utilized validated tools since our primary focus is not to determine the efficacy of various post-ICU interventions. Rather, we strive to understand the configurations in which post-ICU programs and contexts align to produce long-term outcomes.

**Fig. 1:**
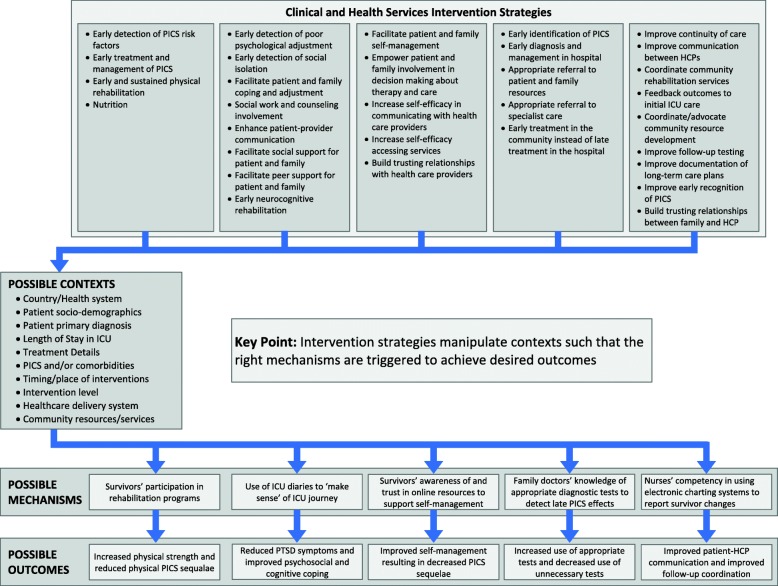
Preliminary program theory

As the second priority of this step, our team identified clinical and health service interventions, which we collated into a preliminary program theory. We initially identified intervention program theory areas in line with Maley and Mikkelsen’s framework as follows: increasing awareness of PICS, educating survivors and caregivers, mitigating risks of physical and neuropsychological impairment, providing access to appropriate services, and coordinating longitudinal care [27]. These theory areas represent potential processes through which clinical or health service interventions improve ICU survivor outcomes. We adapted this framework by incorporating the following concepts or contextual factors to produce our preliminary program theory (Fig. 1): timing and length of the intervention, the target of the intervention in terms of the specific patient characteristics (i.e., original diagnoses, treatments, and specific PICS), the level of the intervention (individual survivor, health care professional, or organization), the healthcare setting (inpatient, outpatient, or mixed), and the health system (according to country).

For this realist review, we are focused on identifying literature that informs the development and refinement of our preliminary program theory. As per Pawson [24], a realist review is non-linear, and the stages of this review process are intricately linked. In keeping with the realist review tradition, the preliminary findings of our literature search guided, and informed, our preliminary program theory. The skeleton for our preliminary program theory will evolve and expand based on the literature and our teams’ engagement in face-to-face and virtual discussions. We will uncover new theories of intervention mechanisms in the literature and refine the intervention theories and concepts/contexts to revise our preliminary program theory, which will serve as the basis for the data extraction forms.

### Step 2: Searching for evidence

To build an understanding of program theories relevant to post-ICU care, we will conduct an expansive multi-database search and iteratively screen the results. Through this process, we will iteratively build upon our search using citation searching and snowballing search strategies to identify additional key documents. We will also purposely search the gray literature to address gaps and supplement our developing program theory.

#### Database search

In consultation with our Clinical Librarian and Information Specialist team member (AR), we developed keyword and subject heading search strings for the population, intervention, and outcome components of our research questions. Keywords and subject headings within each component were primarily combined using the Boolean operator “or” and then the Boolean operator “and” was used to combine the population, intervention, and outcome search strings. Advanced combinations of “or” and “and” Boolean operators were utilized within component groups to intentionally search for specific bodies of literature (e.g., severe AND sepsis). Usage of such combinations balanced feasibility with specificity. Our initial search strategy was constructed for the MEDLINE (via Ovid) database. This search strategy was then adapted to three additional databases: CINAHL (via EBSCO), PsycINFO (via EBSCO), and EMBASE (via Ovid). We limited our searches to English language with no date restriction. Once the search strings for all four databases were finalized using an iterative search and review process, the searches were run consecutively on April 30, 2018 (see Additional file 1: Appendix A for MEDLINE search strategy). These searches generated 6672 citations.

#### Study selection and screening

In a traditional realist review methodology, searching and screening citations occur iteratively to generate or refine theory elements and to explore aspects of a process of interest [28]. In this review, we will amalgamate citations from the four databases mentioned above to build an expansive collection of research relevant to our research questions and objectives. All additional searching and subsequent screening will purposely address specific theoretical hypotheses.

We downloaded all metadata provided by the databases for each of the 6672 citations (authors, title, abstract, keywords, etc.) and imported the citations into ENDNOTE, a reference management program, for duplicate screening. Automated duplicate screening was performed using the built-in ENDNOTE duplicate screening tool, which was set to use title, authors, year, and publication type metadata to identify duplicate citations. Manual duplicate screening was also performed to identify any remaining duplicates. Duplicate screening resulted in a set of 5409 citations, which we imported into the data management software program, EPPI Reviewer 4™, for relevance screening.

We randomly assigned the 5409 citations into ten groups for relevance screening. A preliminary screening protocol was developed that delineated inclusion criteria for population, intervention, and outcome components as described above. This protocol was trialed by one team member (WH) for the first randomized group. A team meeting was held to review the initial results from the preliminary screening, to resolve questions regarding the application of initial screening inclusion criteria, and to adjust—or create additional—screening criteria to address unanticipated citations. Due to the iterative nature of realist approach, we modified our screening protocol as we refined the inclusion criteria. A working version of our screening protocol is depicted in Additional file 1: Appendix B. The EPPI Reviewer 4™ has built-in functionality that enables the reviewers to identify citations that are affected by screening protocol changes. Consequently, in line with the realist review methodology, our screening process will be iterative and will allow for ongoing screening protocol changes. We will not select reviews for data extraction; however, this relevant body of literature will be accessible and organized in the EPPI Reviewer 4™. We will consult this body of literature throughout the review process to enhance our understanding of underlying ideas, assumptions, and theories of post-ICU interventions, as per the RAMESES publication standards for realist review [28].

### Step 3: Extracting data and appraising primary studies

We will import the full-text of all articles screened as “relevant” into the EPPI Reviewer 4™ in preparation for data extraction and study appraisal. We will perform two phases of data extraction; the first will be a matrix method data extraction strategy to understand the landscape of post-ICU intervention studies and the second will employ a theory-driven data extraction strategy. We will adapt a standardized data extraction form and translate the form into EPPI Reviewer 4™ codesets, enabling us to extract preliminary data regarding study design, population, context, interventions, and outcomes. This data will contribute to the selection and appraisal of documents for the theory-driven data extraction.

The aim of the theory-driven data extraction is to refine our program theory using evidence. A unique feature of realist review is that, unlike traditional systematic reviews, a bespoke set of data extraction forms are developed based on the content of the developing program theory [29]. We will develop an extraction tool following Pawson’s recommendations [25], house the data extraction tool on EPPI Reviewer 4™, and pre-test the usability and functionality of our data extraction tool to promote a consistent approach. All team members will extract the data from two purposefully selected articles, and modifications to the tool will be made according to team input. Two research team members (FH and WH) will assume primary responsibility for data extraction. We will review each source independently, share the results, and engage in collaborative decision-making using the project objectives as a guide to collate the information.

In a realist review, the units of analysis are the theories underpinning the interventions (or families of interventions), rather than the interventions per se. Interventions manipulate contexts such that the correct mechanisms are triggered to produce the desired outcomes [26]. During this phase of data extraction, we will extract details about intervention families so that we will be able to organize program theories and CMO configurations by intervention families. The primary focus of this phase of data extraction will be identifying data for the purpose of testing mechanism hypotheses to explore the contexts in which specific mechanisms produce particular outcomes, that is context, mechanism, and outcome configurations. Thus, we will read and index passages of the full-text of documents within EPPI Reviewer 4 to gather information on the type of data (descriptive vs. explanatory), data configurations (contexts, mechanisms, and outcomes of interests), theory type (mid-range and program theories), and methodological rigor. Rigor appraisal will include an evaluation of each study’s methods and analysis and a decision regarding whether or not the inferences drawn from the original study have sufficient weight to make a methodologically credible contribution to the test of the program theory [28]. In contrast to traditional systematic reviews that use checklists to judge the quality and inclusion/exclusion of original studies, we will identify the ways in which the methods (considering issues of sample size, data collection, data analysis, and claims made) influence study conclusions and inherent limitations of each study [25]. We will also consider how documents may contribute to our program theories using the “inference to the best explanation” approach since it will enable us to account for varied trustworthiness within the data [30]. This approach judges the coherence of a program theory by examining (1) the extent to which the theory can explain the data, (2) the simplicity of the theory, and (3) the congruency of the theory to the data and substantive theory [30]. By adopting this approach, we will expand our ability to build and refine the arguments that underpin our program theories.

### Step 4: Synthesizing evidence and sharing conclusions

The basic task of the synthesis process will be to refine our intervention/program theory, that is, to determine how programs (clinical and health services) improve post-ICU patient outcomes, for whom do they work, how they work, and in what circumstances. We will follow the synthesis steps outlined by Rycroft-Malone et al. [29] and organize and manage this synthesis via the EPPI Reviewer 4™. In the first step of synthesis, we will organize the data in the data extraction table into theory and research question areas. For example, we will create a table specifically related to the question how the interaction between patient characteristics and post-ICU interventions affect patient outcomes, which would include all extracts of data and the link back to the source papers. Step two will involve coding the data and identifying and categorizing context, mechanism, and outcome details using an inductive approach, similar to a qualitative thematic analysis. Step three will involve interrogating this data for connections and relationships across data and themes to build up a cumulative picture of potential contexts, mechanisms, and outcome configurations. Finally, we will form hypotheses (contexts, mechanisms, outcomes, and relationships among themes) that can be linked back to source evidence. These hypotheses will act as synthesizing statements of the findings that will be embedded within our program theories. The findings will be written as a narrative according to the RAMESES publication standards [28].

### Step 5: Disseminating and implementing recommendations

In order to meet the practical needs of our knowledge user team members and to maintain methodologic congruence as per a realist approach, validation of emergent findings and the final synthesis and conclusions will involve our entire team. We will develop the preliminary results of this realist review in the form of a report with findings grouped according to each of the above-mentioned hypotheses, which will summarize the nature of the mechanisms, context and outcome links, and the characteristics of the evidence underpinning them. This will include a discussion of the overall strengths and limitations of the knowledge base in post-ICU intervention studies, the quantity and characteristics of published studies, questions addressed, and an assessment of key issues that have not been adequately researched. We will hold a meeting with all team members to determine if the synthesis results capture data relevant to the research questions and address knowledge user team members’ needs or if further data extraction and/or analysis is warranted.

The information gathered and evidence produced from our research will be made available in real time to our knowledge user team members. This will enable them to use the realist review outcomes immediately to develop evidence-based health solutions for ICU survivors in their organization. Our knowledge users will lead the process of integrating the findings into strategic planning in their organization to inform health services development for post-ICU survivors. We will develop communication tools as a team that include one-page documents with bullet points of the key review messages, a two-page executive summary, a PowerPoint presentation, and one or two additional user-friendly communication tools. All materials will be available online and in print. This process has been tested and refined by Suter et al., who recently completed a knowledge synthesis on health systems integration [31]. We will also disseminate our review findings via national and international conferences, as well as peer-reviewed journal publications, in order to make theoretical and empirical contributions to ICU survivorship and develop a more robust knowledge base for applied research.

## Discussion

This protocol outlines our team’s approach to conducting a realist review to create the theoretical scaffolding to support the development of post-ICU care in British Columbia, Canada. The focus of this research is to develop a program theory, encompassing the contexts, mechanisms, and outcomes, to explain how clinical and health service interventions to improve ICU survivor outcomes operate in different contexts for different survivors, and with what effect. Our team of researchers, knowledge users (clinicians and healthcare administrators), and a health science librarian will work collaboratively throughout the process of this realist review, thereby ensuring that we generate theory that meets knowledge user needs and facilitating the implementation of review findings into health service development. Forming a realist-based theory of post-ICU interventions will advance scholarly knowledge, which is also essential for knowledge users internationally, who are increasingly tasked with identifying community interventions that will accelerate recovery and improve long-term outcomes after critical illness.

## Additional file

Additional file 1: Appendix A MEDLINE (via Ovid) Search Strategy. Appendix B Screening Protocol Working Version (DOCX 35 kb)

## Abbreviations

CMO: Contexts, mechanisms and outcomes
ICU: Intensive care unit
PICS: Post-intensive care syndrome
