# Supplementary material for: Health solutions to improve post-intensive care outcomes: a realist review protocol
Source: Syst Rev. 2019 Jan 8;8:11. doi: 10.1186/s13643-018-0939-7 (PMC6323758; doi:10.1186/s13643-018-0939-7)
Supplement: Supplementary file 1 — Appendix A MEDLINE (via Ovid) Search Strategy. Appendix B Screening Protocol Working Version (DOCX 35 kb) [file 13643_2018_939_MOESM1_ESM.docx]

**Additional file 1**

**Appendix A: MEDLINE (via Ovid) Search Strategy**

| **Step** | **Search** |
| --- | --- |
| 1 | Intensive Care Units/ |
| 2 | Critical Care/ |
| 3 | ICU.tw. |
| 4 | CCU.tw. |
| 5 | intensive care.tw. |
| 6 | critical care.tw. |
| 7 | 1 or 2 or 3 or 4 or 5 or 6 |
| 8 | Surviv*.tw. or Survivors/ |
| 9 | (Post Discharge or post-discharge or discharged).tw. |
| 10 | (Post Rehabilitation or post-rehabilitation).tw. |
| 11 | (Post-intensive or postintensive or post-icu).tw. |
| 12 | 8 or 9 or 10 or 11 |
| 13 | Critical Illness/ |
| 14 | (Critically Ill or Critical Illness).tw. |
| 15 | (13 or 14) |
| 16 | Exp respiration, artificial/ |
| 17 | (Decannulat* or mechanically ventilated).tw. |
| 18 | Respiratory Distress Syndrome, Adult/ |
| 19 | (acute respiratory distress syndrome or ARDS).tw. |
| 20 | (16 or 17 or 18 or 19) |
| 21 | sepsis/ or bacteremia/ or endotoxemia/ or hemorrhagic septicemia/ or fungemia/ or candidemia/ or shock, septic/ |
| 22 | (Sepsis or septic).tw. |
| 23 | severe.tw |
| 24 | (21 or 22) AND 23 |
| 25 | extracorporeal membrane oxygenation/ |
| 26 | (extracorporeal membrane oxygenation or ECMO).tw. |
| 27 | (25 or 26) |
| 28 | (12 AND 15) or (12 AND 20) or (12 AND 24) or (12 AND 27) |
| 29 | 7 or 28 |
| 30 | (follow-up or follow up or followup).tw |
| 31 | ambulatory care facilities/ or community health centers/or outpatient clinics, hospital/ |
| 32 | exp "Continuity of Patient Care"/ |
| 33 | exp Physical Therapy Modalities/ |
| 34 | rehabilitation.tw. |
| 35 | Critical Illness/rh |
| 36 | Aftercare.tw. |
| 37 | (journal* or diar*).tw |
| 38 | recovery.tw |
| 39 | program*.tw |
| 40 | 38 and 39 |
| 41 | 30 or 31 or 32 or 33 or 34 or 35 or 36 or 37 or 40 |
| 42 | 31 or 32 or 33 or 34 or 35 or 36 or 37 or 40 |
| 43 | Mental Health/ |
| 44 | exp Mental Disorders/ |
| 45 | exp Memory Disorders/ |
| 46 | affective symptoms/ or aggression/ or catatonia/ or delusions/ or depersonalization/ or depression/ or mental fatigue/ or stress, psychological/ |
| 47 | Muscle Weakness/ |
| 48 | (Post-intensive or postintensive or post-icu).tw. |
| 49 | Anxiety.tw. |
| 50 | Depression.tw. |
| 51 | Memory.tw. |
| 52 | Post-traumatic stress.tw. |
| 53 | PTSD.tw. |
| 54 | PICS.tw. |
| 55 | Intensive.tw. |
| 56 | 54 and 55 |
| 57 | Critical illness.tw. |
| 58 | Discharg*.tw. |
| 59 | 57 and 58 |
| 60 | exp "OUTCOME ASSESSMENT (HEALTH CARE)"/ |
| 61 | outcome*.tw |
| 62 | exp morbidity/ or mortality/ or "cause of death"/ or survival rate/ |
| 63 | "Quality of Life"/ |
| 64 | 60 or 61 or 62 or 63 |
| 65 | longterm.tw |
| 66 | long-term.tw |
| 67 | (Post Discharge or post-discharge or discharged).tw. |
| 68 | (Post-intensive or postintensive or post-icu).tw. |
| 69 | "after discharge".tw |
| 70 | "following discharge".tw |
| 71 | 65 or 66 or 67 or 68 or 69 or 70 |
| 72 | 64 and 71 |
| 73 | 43 or 44 or 45 or 46 or 47 or 48 or 49 or 50 or 51 or 52 or 53 or 56 or 59 or 72 |
| 74 | 43 or 44 or 45 or 46 or 47 or 48 or 49 or 50 or 51 or 52 or 53 or 56 or 59 |
| 75 | (29 AND 41 AND 74) OR (29 AND 42 AND 73) |
| 76 | limit 75 to english language |
| 77 | (pediatric* or paediatric* or neonat*).tw. |
| 78 | 76 not 77 |

**Appendix B: Screening Protocol Working Version**

| **Step** | **Process** |
| --- | --- |
| Step 1: Population/Discussion Screening | Perform ‘Population/Discussion Screening. If ‘Relevant’, code as ‘Relevant’ and continue to ‘Intervention Screening’. If ‘Not Relevant’, code as ‘Not Relevant’, exclude, and consider for background. If you encounter a new population is comparable to general ICU patients and is unaccounted for, code as 'Unsure', and use the ‘Info’ text box function to describe the population. Then proceed to ‘Intervention Screening’. You can also use this textbox to document and explain why you screened an article as ‘Unsure’ for the intervention and outcome screening components. |
| Step 2: Intervention Screening | Perform ‘Intervention Screening’. If 'Relevant', code as ‘Relevant’ and continue to ‘Outcome Screening’. If ‘Not Relevant’, code as ‘Not Relevant’, exclude and consider for background. If ‘Unsure,’ code as ‘Unsure’ and proceed to ‘Outcome Screening’. |
| Step 3: Outcome Screening | Perform ‘Outcome Screening’. If 'Relevant', code as ‘Relevant’ unless one or two of the previous component screenings resulted in an 'Unsure' code. If any of the previous screenings resulted in an ‘Unsure’ code, exclusively code as 'Unsure'. If Outcome Screening results in ‘Unsure’ code, code as ‘Unsure’. If Outcome Screening results in 'Not Relevant' code, code as ‘Not Relevant’, exclude, and consider for background. |

| **STEP 1: Population/Discussion Screening** | | |
| --- | --- | --- |
|  |  |  |
| **Population** | **Screen As** | **Then** |
| Pop = Majority or Exclusively General ICU Pts | Relevant | INT SC |
| Pop = Surgical ICU Pts | Relevant | INT SC |
| Pop = Pulmonary ICU Pts | Relevant | INT SC |
| Pop = Caregivers/family of ICU Pts | Relevant | INT SC |
| Pop = Cystic fibrosis | Relevant If ICU Stay | INT SC |
| Pop = Frail/Elderly | Relevant If ICU Stay | INT SC |
| Pop = IVDU | Relevant If ICU Stay | INT SC |
| Pop = Pts with pre-existing conditions | Relevant If ICU Stay | INT SC |
| Pop = Severe necrotizing soft-tissue infections | Relevant If ICU Stay | INT SC |
| Pop = Cardiac Pts | Relevant If ICU Stay | INT SC |
| Pop = Cardiac Rehab Pts | Relevant If ICU Stay | INT SC |
| Pop = Surgery Pts | Relevant If ICU Stay | INT SC |
| Pop = Surgical Rehab Pts | Relevant If ICU Stay | INT SC |
| Pop = Pulmonary/Respiratory Pts | Relevant If ICU Stay | INT SC |
| Pop = Pulmonary Rehab Pts | Relevant If ICU Stay | INT SC |
| Pop = Burn Pts | Not Relevant | EX;CFB |
| Pop = Cancer Pts | Not Relevant | EX;CFB |
| Pop = Transplant Pts | Not Relevant | EX;CFB |
| Pop = Coronary (Care) Unit Pts | Not Relevant | EX;CFB |
| Pop = Cardiac (Surgery) ICU Pts | Not Relevant | EX;CFB |
| Pop = Psychiatric ICU Pts | Not Relevant | EX;CFB |
| Pop = Neurointensive care unit Pts | Not Relevant | EX;CFB |
| Pop = Neurotrauma (Spinal Cord/Head) | Not Relevant | EX;CFB |
| Pop = ABI/TBI/Stroke Pts | Not Relevant | EX;CFB |
| Pop = Neurorehabilitation Pts | Not Relevant | EX;CFB |
| Pop = Not Adults (E.g. Infants or Children) | Not Relevant | EX;CFB |
| Pop = Health Care Providers | Not Relevant | EX;CFB |
| **Excluded Discussions** | **Screen As** | **Then** |
| Ethical Discussions | Not Relevant | EX;CFB |
| End of Life Care | Not Relevant | EX;CFB |
| Protocol Manuscripts | Not Relevant | EX;CFB |
| Reviews | Not Relevant | EX;CFB |
| Case Reports | Not Relevant | EX;CFB |
| Tool Development/Evaluation/Validation | Not Relevant | EX;CFB |
| No Evidence of ICU Stay | Not Relevant | EX;CFB |
| Conferences and Abstracts | Not Relevant | EX;CFB |
| **Included Discussions** | **Screen As** | **Then** |
| ICU follow-up clinics/rehab (1° Studies) | Relevant | Code for DE |
| **Unsure Populations** | **Screen As** | **Then** |
| Pop = Maternal/Pregnant/Post-Partum | Unsure | INT SC |
| Pop = Intermediate care unit patients | Unsure | INT SC |

| **STEP 2: Intervention Screening** | | |
| --- | --- | --- |
|  |  |  |
| **Studies With Interventions** | **Screen As…** | **Then** |
| Intervention Timing = Post-Hospital Discharge (Begins in, or continues into post-hospital period) | Relevant | OUT SC |
| Intervention Timing = Unclear | Unsure | OUT SC |
| Intervention Timing = Pre-Hospital Discharge | Not Relevant | EX;CFB |
| **Studies Without Interventions** | **Screen As…** | **Then** |
| Studies with no apparent intervention or studies that do not manipulate a post-hospital intervention. E.g. Prognostic, Retrospective, Descriptive, Identification Studies, ...etc. | Screen for post-hospital discharge interventions or management strategies [IoMS] in the title and abstract. If there is evidence of an IoMS, then screen for data collection timing. If data collection regarding the IoMS occurs post-ICU discharge, then screen as 'Relevant'; If unclear, screen as ‘Unsure’. Otherwise, screen as 'Not Relevant'. | |
|  |  |  |
|  |  |  |
|  |  |  |
|  |  |  |
|  |  |  |
| No discussion of Post-Discharge IoMS | Not Relevant | EX;CFB |
|  |  |  |
| **STEP 3: Outcome Screening** | | |
|  |  |  |
| **Criteria** | **Screen As…** | **Then** |
| Outcomes or Experiences ≥ 28 days | Relevant | Code for DE |
| Outcome/Experience measurement time unclear | Unsure | Screen Full Text |
| Outcome or Experiences ≤ 28 days | Not Relevant | EX;CFB |

| **Abbreviations** | **Meaning** |
| --- | --- |
| INT SC | Intervention Screening |
| OUT SC | Outcome Screening |
| EX | Exclude |
| DE | Data Extraction |
| CFB | Consider For Background |
| IoMS | Intervention or Management Strategy |
| 1° | Primary |
| ICU | Intensive Care Unit |
| Pts | Patients |
| ABI | Acute Brain Injury |
| TBI | Traumatic Brain Injury |
| IVDU | Intravenous Drug User |
